# Geographical Characterization of Tunisian Olive Tree Leaves (cv. Chemlali) Using HPLC-ESI-TOF and IT/MS Fingerprinting with Hierarchical Cluster Analysis

**DOI:** 10.1155/2018/6789704

**Published:** 2018-03-13

**Authors:** Amani Taamalli, David Arráez Román, Ana María Gómez Caravaca, Mokhtar Zarrouk, Antonio Segura Carretero

**Affiliations:** ^1^Laboratoire de Biotechnologie de l'Olivier, Centre de Biotechnologie de Borj-Cedria, Hammam-Lif, Tunisia; ^2^Center of Research and Development of Functional Food, Health Science Technological Park, Avda. del Conocimiento s/n, 18100 Granada, Spain; ^3^Department of Analytical Chemistry, Faculty of Sciences, University of Granada, 18071 Granada, Spain

## Abstract

The olive plant has been extensively studied for its nutritional value, whereas its leaves have been specifically recognized as a processing by-product. Leaves are considered by-products of olive farming, representing a significant material arriving to the olive mill. They have been considered for centuries as an important herbal remedy in Mediterranean countries. Their beneficial properties are generally attributed to the presence of a range of phytochemicals such as secoiridoids, triterpenes, lignans, and flavonoids. With the aim to study the impact of geographical location on the phenolic compounds, *Olea europaea* leaves were handpicked from the Tunisian cultivar “Chemlali” from nine regions in the north, center, and south of Tunisia. The ground leaves were then extracted with methanol : water 80% (*v*/*v*) and analyzed by using high-performance liquid chromatography coupled to electrospray time of flight and ion trap mass spectrometry analyzers. A total of 38 compounds could be identified. Their contents showed significant variation among samples from different regions. Hierarchical cluster analysis was applied to highlight similarities in the phytochemical composition observed between the samples of different regions.

## 1. Introduction

Natural antioxidants present in the diet have evidenced the increase of the resistance toward oxidative damages, and they may have a substantial impact on human health [1]. Furthermore, the use of natural extracts from plants as functional ingredients in food, beverage, and cosmetic applications is receiving a great deal of attention among scientists, as well as among consumers and food manufacturers [2]. Recently, there has been a renewed interest in natural product research due to the failure of alternative drug discovery methods to deliver many lead compounds in key therapeutic areas such as immune suppression, anti-infectives, and metabolic diseases [3]. Despite competition from other drug discovery methods, natural products are still providing their fair share of new clinical candidates and drugs [3]. We can find several natural products as sources of new drugs that have been recently reviewed in literature [4–7].

The olive plant has been extensively studied for its nutritional value, whereas its leaves have been specifically recognized as a processing by-product [8]. Considered as by-products of olive farming, olive tree leaves represent a significant material arriving to the olive mill [9]. They have been considered for centuries as an important herbal remedy in Mediterranean countries. Their positive effects on human health are multiple: antioxidant [10–16], hypoglycemic [17], antimicrobial [18], and anticancerous [19–22] among others. These properties are generally attributed to the presence of a range of compounds, such as secoiridoids, triterpenes, lignans, and flavonoids. It has been reported that by-products can have a similar or even higher proportion of bioactive compounds than the usable parts of produce [23]. For instance, the characterization and quantification of bioactive compounds is one of the first steps to be taken in any evaluation of the putative contribution of the olive and its derivatives to human health [24] since the chemical composition of olive tree leaves varies depending on several conditions such as origin, proportion of branches on the tree, storage conditions, climatic conditions, moisture content, and degree of contamination with soil and oils [25]. Recently, the effects of abiotic and biotic factors on the phenolic composition in olive tree leaves have been reviewed in literature [26]. Biotic factors such as genotype, load bearing, leaves age, and fungi and bacteria attacks as well as abiotic factors such as hydric deficiency, salinity, fertilization, sampling time, geographical zone, and climatic conditions influence the contents of phenolic compounds [26]. Recent researches revealed the impact of the geographical location on the total phenolic composition of olive leaves [27–30]. However, the data on the variation of the individual phenolic compounds in olive leaves are scarce. According to our knowledge, this is the first study on the phenolic compounds in olive tree leaves from Tunisian cultivars according to the geographical region.

Considering the importance of phenolic compounds in olive leaves, their determination and the scarce literature related to their behavior according to geographical origin, the aim of this study was to explore the phenolic composition of the leaves from the Tunisian olive cultivar “Chemlali” from different localities. This cultivar was chosen since it is widespread throughout Tunisia from the north to the south.

## 2. Materials and Methods

### 2.1. Sample Collection and Preparation of Extracts

Fresh olive leaves from the Tunisian “Chemlali” cultivar were obtained at the time of olive pruning from different regions from Tunisia (north, center, and south from coastal and mountainous areas) (available here). Leaves were dried at 25°C, ground, and then 1 g of the obtained homogenized powder was dissolved in 10 mL of methanol : water 80% (*v*/*v*), and the mixture was allowed to stand in the dark for 24 h [31]. The extract was centrifuged at 5000 g for 10 min at room temperature, and the supernatants were then filtered using a 0.45 *µ*m syringe filter to be analyzed by high-performance liquid chromatography (HPLC).

### 2.2. Chromatographic Separation of Phenolic Compounds

An Agilent 1200 HPLC system (Agilent Technologies, Waldbronn, Germany) equipped with a vacuum degasser, autosampler, a binary pump, and a DAD detector was used for the chromatographic determination. Phenolic compounds were separated by using an Agilent Eclipse Plus C18-column (4.6 × 150 mm, 1.8 mm particle size) operating at 25°C and a flow rate of 0.8 mL/min. The mobile phases used were water with acetic acid (0.5%) (phase A) and acetonitrile (phase B), and the solvent gradient changed according to the following conditions: 0 to 10 min, 5–30% B; 10 to 12 min, 30–33% B; 12 to 17 min, 33–38% B; 17 to 20 min, 38–50% B; and 20 to 23 min, 50–95% B. Finally, the B content was decreased to the initial conditions (5%) in 2 min, and the column reequilibrated for 10 min. A volume of 10 mL of the extracts was injected. The separated compounds were monitored in sequence first with a diode-array detector (DAD) at 240 and 280 nm and then with a mass spectrometry (MS) detector.

### 2.3. Time of Flight and Ion Trap Mass Spectrometry Detection

The HPLC system was firstly coupled to a Bruker Daltonics micrOTOF mass spectrometer (Bruker Daltonics, Bremen, Germany) using an orthogonal electrospray interface. The mass spectrometer was operated in the negative ionization mode and acquired data in the mass range from *m*/*z* 50 to 1000 with a spectra rate of 1 Hz. The capillary was set at +4 kV, the end plate offset at −500 V, the nebulizer gas at 2 bar, and the dry gas at 9 L/min at 250°C. The accurate mass data of the molecular ions were processed through the software data analysis and target analysis 4.0 (Bruker Daltonics). External mass spectrometer calibration was performed using a Cole-Parmer syringe pump (Vernon Hills, Illinois, USA) directly connected to the interface, equipped with a Hamilton syringe (Reno, Nevada, USA). The standard solution was injected at the beginning of the run, and all the spectra were calibrated prior to phenolic compounds' identification. Then, the same HPLC system was coupled to a Bruker Daltonics Esquire 2000 ion trap mass spectrometer (Bruker Daltonics, Bremen, Germany) equipped with an electrospray interface (Agilent Technologies, CA, USA) operating in the negative ionization mode. The ion trap scanned at the 50–1000 *m/z* range at 13000 *µ*/s during the separation and detection. The maximum accumulation time for the ion trap was set at 200 ms, the target count at 20000, and compound stability was set at 50%. The optimum values of the ESI-MS parameters were as follows: capillary voltage, +3.0 kV; drying gas temperature, 300°C; drying gas flow, 7.0 L/min; and nebulizing gas pressure, 21.7 psi. The instrument was controlled by Esquire NT software from Bruker Daltonics. Peak identification was performed on the basis of their relative retention time values, TOF-MS and IT-MS/MS data, comparison with authentic standard solutions, and using the information previously reported in the literature [32]. Limits of detection (LOD) and quantification (LOQ) were respectively as follow: 0.09 and 0.3 ppm for hydroxytyrosol, 0.31 and 1.03 ppm for tyrosol, 0.02 and 0.06 ppm for luteolin, and 0.02 and 0.06 ppm for apigenin.

### 2.4. Radical-Scavenging Capacity (RSC)

The olive leaf samples were analyzed for their capacity to scavenge the stable DPPH^•^ radical [33]. A volume of 3 mL of a freshly prepared DPPH solution (10^−4^ M in methanol) was added to 0.5 mL of the polar extract. The reaction mixture was shaken vigorously for 10 s in a vortex apparatus and then maintained in the dark for 20 min, after which a steady state was reached. The absorbance of the mixture was measured at 515 nm against a blank solution (without radical). A control sample (without olive leaf extract) was prepared and measured. The RSC toward DPPH^•^ was expressed as the % reduction in DPPH^•^ concentration by the constituents of the oils: % [DPPH^•^] red = 100∗(1 – [DPPH^•^]_20_/[DPPH^•^]_0_) where [DPPH^•^]_0_ and [DPPH^•^]_20_ were the concentrations of DPPH^•^ in the control sample (*t*=0) and in the test mixture after the 20-minute reaction, respectively.

### 2.5. Statistical Analysis

Statistical analysis was performed by means of XLSTAT for Windows. Data are given as means of triplicates. Hierarchical cluster analysis (HCA) was performed using Ward's method based on Euclidean distance.

## 3. Results and Discussion

### 3.1. HPLC-MS Analysis of the Olive Leaf Extract

**Figure 1:**
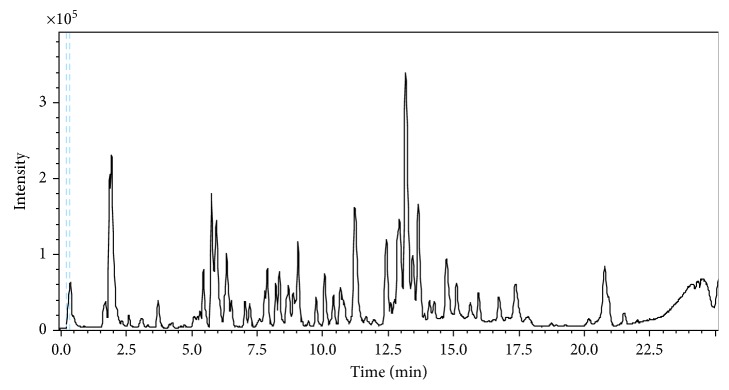
Base peak chromatogram of “Chemlali” olive leaf extract from Zaghouan region.

The base peak chromatogram (BPC) of “Chemlali” olive leaf extract and the compounds characterized in the negative ion mode are shown in Figure 1. Peak identification was carried out using Target Analysis software (Bruker Daltonics), by comparing both migration time and accurate MS and MS/MS spectral data obtained from olive leaf samples and commercial standards, together with the information previously reported in the literature.

**Figure 2:**
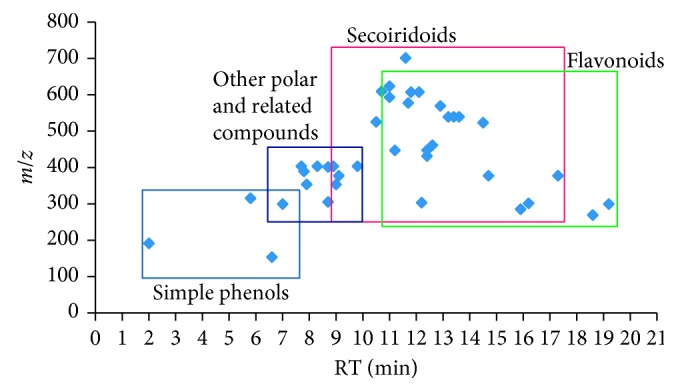
Distribution of the identified compounds according to their retention time and *m/z*.

Table 1 summarizes the information about the identified phenolic compounds, in “Chemlali” olive leaf extracts, together with their corresponding retention time, theoretical mass to charge ratio (*m/z*), molecular formula, and IT/MS^2^ fragments. A total of 38 compounds could be identified. Three main phenolic groups could be clearly distinguished: phenolic alcohols (hydroxytyrosol, tyrosol, and tyrosol glucoside), flavonoids (luteolin rutinoside, 2 isomers of luteolin glucoside, 2 isomers of diosmin, apigenin rutinoside, apigenin glucoside, chryseriol glucoside, luteolin, apigenin, rutin, quercetin, gallocatechin, taxifolin, and diosmetin), and various secoiridoids (particularly, oleuropein with 3 isomers, methoxyoleuropein, ligstroside, oleuropein aglycon derivative and related compounds such as elenolic acid glucoside isomers, and secologanoside). Apart from them, other polar compounds were also found; they are quinic acid, caffeoylquinic acid isomers, and benzyl alcohol hexose-pentose. A certain tendency in the elution order of the compounds related to their chemical structure class was observed, appearing in the following order of increasing retention time (RT), and thus hydrophobicity: simple phenols, secoiridoids and related compounds, and flavonoids (Figure 2).

As can be observed in Table 2, some qualitative differences were registered among olive leaf samples from the geographical studied regions. Quantitatively, significant differences were found in a wide number of phenolic compounds according to the geographical origin of the samples. Our results are in agreement with those reported in literature. In deed comparing Tunisian cultivars located in southern and northern Tunisia, significant differences were observed for phenolic composition as well as antioxidant activity where higher levels were registered for samples from the north [29].

Bilgin and Şahin [28] demonstrated that total phenolic content and extract yield varied significantly among Turkish olive cultivars from six sites in Anatolia. The authors observed that the total phenolic contents in olive leaves decreased as the geographical altitude decreases [28]. In another study on Greek olive cultivars, leaves collected from three different locations showed differences in total phenolic contents and antioxidant activity [27]. Similarly, the geographical zone has influenced the phenolic composition of Arbequina cultivar samples collected from six regions in Spain [30]. Using hierarchical cluster analysis, the latter study showed high similarities between the olive leaf samples from Cordoba and Navarra and both samples from Mallorca, respectively.

**Figure 3:**
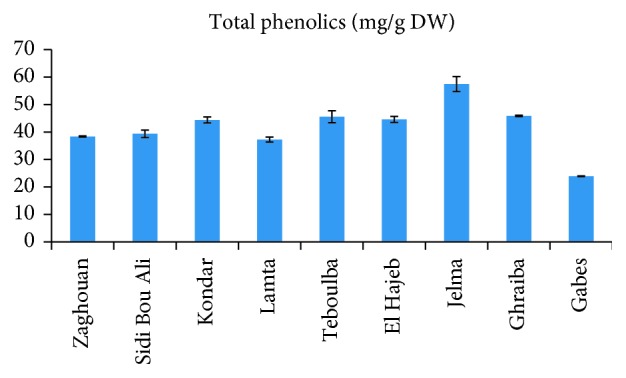
Total phenolic contents in “Chemlali” olive leaf extracts according to the cultivation regions.

**Figure 4:**
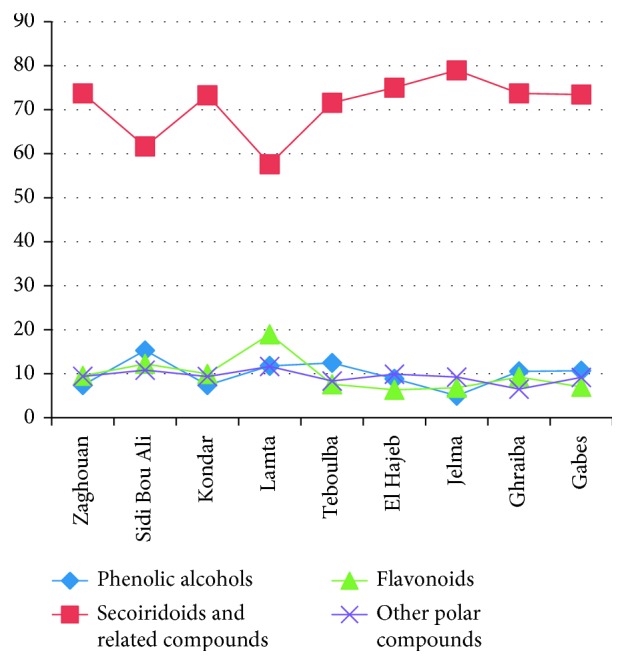
Variation in the distribution of the phenolic compound classes in “Chemlali” olive leaf extracts according to the cultivation regions.

In our study, significant differences were observed for total phenolic contents in “Chemlali” olive leaf extracts from different regions (Figure 3). These differences could be explained by the most marked variation that was observed for flavonoids and secoiridoids and related compounds as well as for phenolic alcohols classes especially for the leaves collected from Lamta region. These classes presented amounts that reached 18, 78 and 15% for Lamta, Jelma, and Sidi Bou Ali, respectively. Furthermore, we can observe that secoiridoids' tendency was the inverse of that of flavonoids (Figure 4).

Being the major one among the identified compounds, oleuropein amounts varied significantly among olive leaf samples. The registered values were between 15.8 and 42.25% for samples from Gabes and Lamta regions, respectively.

**Figure 5:**
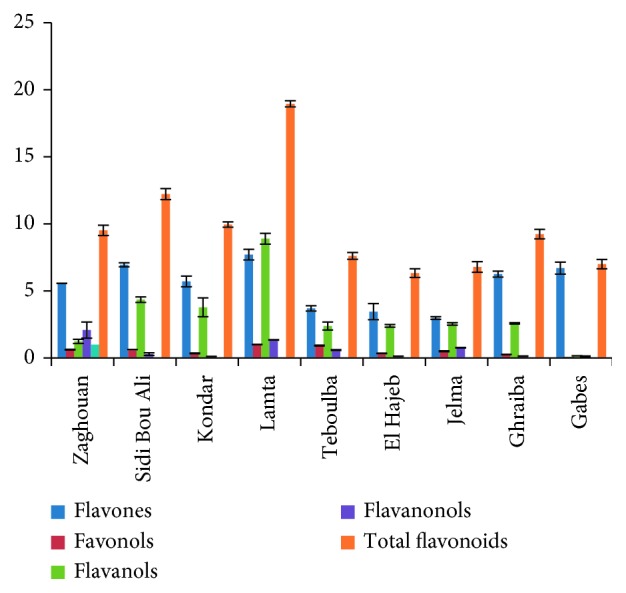
Variation in the distribution of flavonoids in “Chemlali” olive leaf extracts according to the cultivation regions.

**Figure 6:**
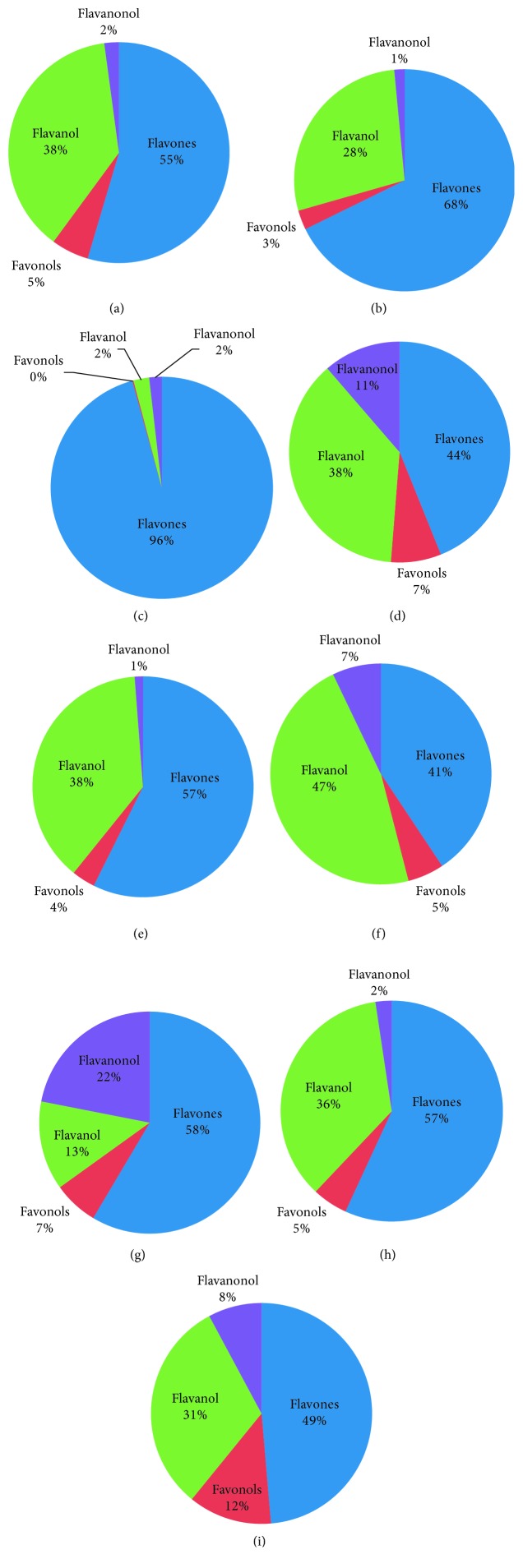
Flavonoid subclasses distribution. (a) El Hajeb. (b) Ghraiba. (c) Gabes. (d) Jelma. (e) Kondar. (f) Lamta. (g) Zaghouan. (h) Sidi Bou Ali. (i) Teboulba.

Considering the subclasses of flavonoids, the most pronounced variation among samples from different regions was registered for flavones and flavanols. Flavones presented the highest percentage among the rest of flavonoids followed by flavanols. Among analyzed samples, olive leaves from Gabes showed the highest percentage in flavones, whereas leaves from Lamta showed the highest levels in flavanols presented by gallocatechin (Figures 5 and 6).

**Figure 7:**
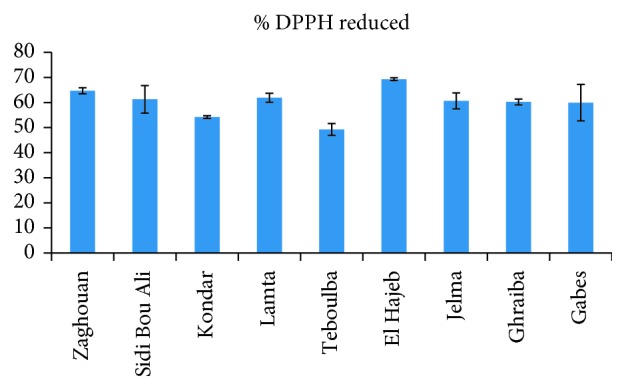
Radical-scavenging capacity of olive leaf extracts according to the cultivation regions.

Radical-scavenging activity is very important, due to the deleterious role of free radicals in foods and in biological systems. This test is a standard assay in antioxidant activity studies and offers a rapid technique for screening the radical-scavenging activity of specific compounds. The stable free DPPH radical is a useful reagent to investigate the scavenger properties of phenolics [34]. The radical-scavenging capacity values varied significantly between studied samples: the leaves from Zaghouan presented the highest radical-scavenging capacity, whilst leaves from Teboulba showed a radical-scavenging capacity of 49% (Figure 7).

### 3.2. Chemometrics

Data clustering is a common technique for statistical data analysis, which is used in many fields. It is known as the classification of objects into groups (clusters), so that the data in each subset share some common trait—often proximity according to some defined distance measure [35]. The hierarchical analysis is a kind of very important clustering methods. It is an unsupervised technique that examines the interpoint distances between all of the samples and represents that information in the form of a two dimensional plot called a hierarchical tree or dendrogram.

In our study, an overall cluster analysis based on the determined metabolites and radical-scavenging capacity was performed to study the general differences among samples. To display the similarities between olive leaf samples from different provenance, a dendrogram was produced by applying a hierarchical clustering algorithm to the data.

**Figure 8:**
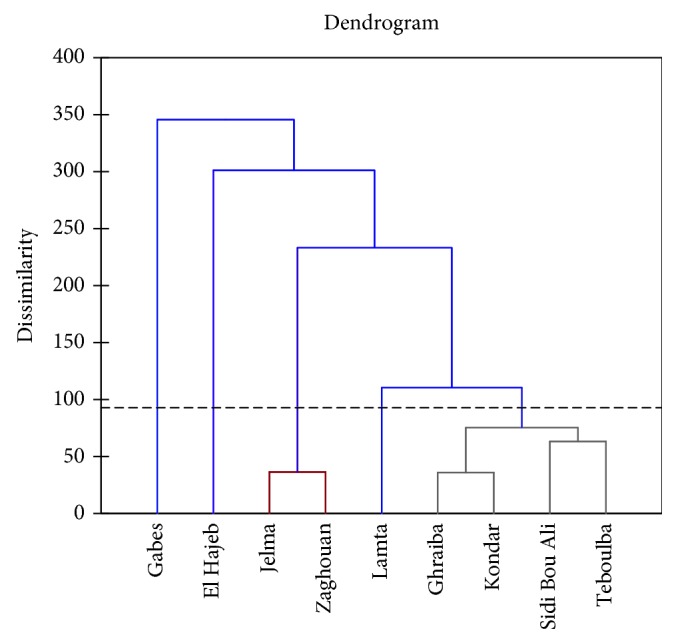
Hierarchical cluster analysis dendrogram. Groups: (1) Gabes; (2) Ghraiba, Kondar, Sidi Bou Ali, Teboulba; (3) El Hajeb; (4) Jelma, Zaghouan; (5) Lamta.

As can be seen in Figure 8, there is a clear separation among the clusters established for different samples. Applying Ward's method based on Euclidean distance, olive leaf samples could be gathered into five different groups with respect to their phenolic composition: the first presented by Gabes region; the second by Ghraiba, Kondar, Sidi Bou Ali, and Teboulba; and the third by El Hajeb. The fourth group is composed by Jelma and Zaghouan and the fifth by Lamta.

**Figure 9:**
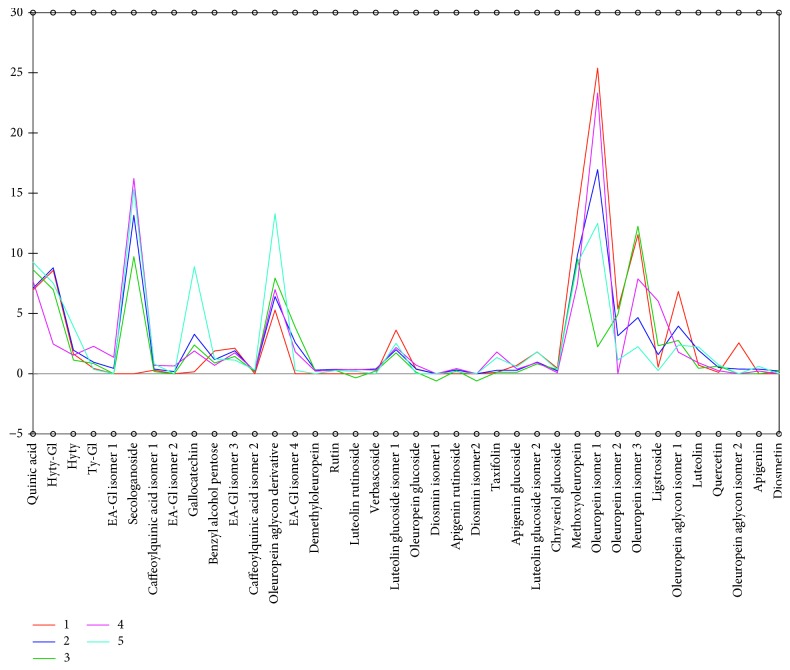
HCA groups' profiles according to the phenolic composition. Groups: (1) Gabes; (2) Ghraiba, Kondar, Sidi Bou Ali, Teboulba; (3) El Hajeb; (4) Jelma, Zaghouan; (5) Lamta.

It is useful to mention that HCA could be successfully applied for the classification and evaluation of the regional growing effects on the specification of the olive leaves based on their phenolic profile. In Figure 9, the profiles of the distinguished clusters or groups are illustrated. As can be seen, the leaves from Lamta region located in the coastal center of Tunisia are richer in gallocatechin and oleuropein aglycone derivative. Groups 1 and 4 presented the leaves richer in oleuropein isomer 1. Moreover, leaves from Gabes (group 1) located in the coastal south of Tunisia showed their richness in oleuropein aglycone.

These results are important since they may be useful for further researches and give an insight into the behavior of phenolic compounds in olive leaves from different environments. Such compounds have shown to be sensible to the edaphoclimatic variation between the considered regions of cultivation of “Chemlali” cultivar.

## Figures and Tables

**Table 1:** Identification data for the compounds detected in olive leaf extracts.

*m/z* [M-H]^−^	RT (min)	Formula	Compound	IT/MS^2^ fragments	Class
191.0561	2.0	C_7_H_12_O_6_	Quinic acid	103	Other polar compounds
315.1085	5.8	C_14_H_20_O_8_	Hydroxytyrosol-hexose	123, 153	Simple phenols
153.0557	6.6	C_8_H_10_O_3_	Hydroxytyrosol	123	Simple phenols
299.1136	7.0	C_14_H_20_O_7_	Tyrosol glucoside		Simple phenols
403.1246	7.7	C_17_H_24_O_11_	Elenolic acid glucoside isomer 1		Secoiridoids and related compounds
389.1089	7.8	C_16_H_22_O_11_	Secologanoside	183, 209, 227	Secoiridoids and related compounds
353.0878	7.9	C_16_H_18_O_9_	Caffeoylquinic acid isomer 1	191	Other polar compounds
403.1246	8.3	C_17_H_24_O_11_	Elenolic acid glucoside isomer 2		Secoiridoids and related compounds
305.0667	8.7	C_15_H_14_O_7_	Gallocatechin	225	Flavanol
401.1453	8.7	C_18_H_26_O_10_	Benzyl alcohol hexose-pentose	161	Other polar compounds
403.1246	8.9	C_17_H_24_O_11_	Elenolic acid glucoside isomer 3	179, 223, 371	Secoiridoids and related compounds
353.1606	9.0	C_16_H_18_O_9_	Caffeoylquinic acid isomer 2	191	Other polar compounds
377.1453	9.1	C_16_H_26_O_10_	Oleuropein aglycon derivative		Secoiridoids and related compounds
403.1246	9.8	C_17_H_24_O_11_	Elenolic acid glucoside isomer c		Secoiridoids and related compounds related compounds
525.1614	10.5	C_24_H_30_O_13_	Demethyloleuropein		Secoiridoids and related compounds
609.1461	10.7	C_27_H_30_O_16_	Rutin	300	Flavonol
593.1512	11.0	C_27_H_30_O_15_	Luteolin rutinoside	285	Flavone
623.2075	11.0	C_29_H_36_O_15_	Verbascoside	461, 315	Secoiridoids and related compounds
447.0933	11.2	C_21_H_20_O_11_	Luteolin glucoside isomer 1	285	Flavone
701.2298	11.6	C_31_H_42_O_18_	Oleuropein glucoside	539, 377, 307	Secoiridoids and related compounds
607.1668	11.8	C_28_H_32_O_15_	Diosmin isomer 1	299	Flavone
577.1563	11.7	C_27_H_30_O_14_	Apigenin rutinoside	269	Flavone
607.1668	12.1	C_28_H_32_O_15_	Diosmin isomer 2	299	Flavone
303.051	12.2	C_15_H_12_O_7_	Taxifolin		Flavanonol
431.0984	12.4	C_21_H_20_O_10_	Apigenin glucoside	268	Flavone
447.0933	12.4	C_21_H_20_O_11_	Luteolin glucoside isomer 2	285	Flavone
461.1089	12.6	C_22_H_22_O_11_	Chrysoeriol glucoside		Flavone
569.1876	12.9	C_26_H_34_O_14_	Methoxyoleuropein	537, 403, 337	Secoiridoids and related compounds
539.1770	13.2	C_25_H_32_O_13_	Oleuropein isomer 1	275, 377, 307	Secoiridoids and related compounds
539.1770	13.4	C_25_H_32_O_13_	Oleuropein isomer 2	275, 377, 307	Secoiridoids and related compounds
539.177	13.6	C_25_H_32_O_13_	Oleuropein isomer 3	275, 377, 307	Secoiridoids and related compounds
523.1820	14.5	C_25_H_32_O_12_	Ligstroside	291, 259	Secoiridoids and related compounds
377.1242	14.7	C_19_H_22_O_8_	Oleuropein aglycon isomer 1		Secoiridoids and related compounds
285.0405	15.9	C_15_H_10_O_6_	Luteolin	175, 151	Flavone
301.0354	16.2	C_15_H_10_O_7_	Quercetin		Flavonol
377.1242	17.3	C_19_H_22_O_8_	Oleuropein aglycon isomer 2	275	Secoiridoids and related compounds
269.0455	18.6	C_15_H_10_O_5_	Apigenin	225, 151	Flavone
299.0561	19.2	C_16_H_12_O_6_	Diosmetin		Flavone

**Table 2:** Phenolic composition (in % of total phenolics) of “Chemlali” olive leaves from different regions.

	Gabes	Ghraiba	El Hajeb	Jelma	Kondar	Lamta	Sidi Bou Ali	Teboulba	Zaghouan
Quinic acid	6.96 ± 0.05	5.21 ± 0.01	8.65 ± 0.45	7.86 ± 0.41	7.82 ± 0.51	9.26 ± 0.36	8.83 ± 0.79	6.58 ± 0.24	7.55 ± 1.02
Hyty-Gl	8.59 ± 0.63	7.05 ± 0.16	6.98 ± 0.21	3.02 ± 0.15	5.13 ±	7.52 ± 0.21	12.96 ± 0.80	9.06 ± 0.37	1.75 ± 0.15
Hyty	1.65 ± 0.02	2.24 ± 0.02	1.01 ± 0.02	1.16 ± 0.01	1.83 ± 0.13	3.90 ± 0.06	1.56 ± 0.08	2.00 ± 0.09	1.92 ± 0.01
Ty-Gl	0.44 ± 0.03	1.24 ± 0.11	0.86 ± 0.07	0.82 ± 0.02	0.45 ± 0.13	0.36 ± 0.15	0.79 ± 0.21	1.39 ± 0.1	3.74 ± 0.33
EA-Gl isomer 1	nd	0.58 ± 0.03	nd	1.39 ± 0.01	0.49 ± 0.1	nq	nd	0.70 ± 0.02	1.34 ± 0.11
Secologanoside	nd	15.78 ± 0.21	9.73 ± 0.29	18.27 ± 0.87	14.36 ± 0.66	15.32 ± 0.81	9.72 ± 0.57	12.77 ± 0.78	14.17 ± 0.53
Caffeoylquinic acid isomer 1	0.30 ± 0.01	0.40 ± 0.08	0.18 ± 0.03	0.60 ± 0.1	0.32 ± 0.08	0.82 ± 0.06	0.42 ± 0.03	0.38 ± 0.02	0.74 ± 0.02
EA-Gl isomer 2	nd	nd	nd	0.95 ± 0.13	nd	nd	nd	0.07 ± 0.01	0.35 ± 0.07
Gallocatechin	0.16 ± 0.01	2.58 ± 0.12	2.39 ± 0.1	2.54 ± 0.01	3.78 ± 0.4	8.89 ± 0.3	4.35 ± 0.24	2.39 ± 0.35	1.24 ± 0.43
Benzyl alcohol hexose-pentose	1.90 ± 0.04	0.70 ± 0.02	0.90 ± 0.01	0.54 ± 0.11	1.03 ± 0.31	1.23 ± 0.27	1.35 ± 0.45	1.26 ± 0.2	0.86 ± 0.11
EA-Gl isomer 3	2.13 ± 0.03	1.90 ± 0.00	1.44 ± 0.01	1.76 ± 0.07	2.49 ± 0.38	1.14 ± 0.13	1.77 ± 0.47	1.42 ± 0.29	1.70 ± 0.19
Caffeoylquinic acid isomer 2	nd	0.20 ± 0.01	0.17 ± 0.01	0.24 ± 0.01	0.19 ± 0.05	0.33 ± 0.01	0.21 ± 0.00	0.13 ± 0.02	0.25 ± 0.02
Oleuropein aglycon derivative	5.29 ± 0.06	7.96 ± 0.03	7.95 ± 0.39	6.83 ± 0.13	10.42 ± 0.81	13.30 ± 0.59	8.85 ± 0.77	7.42 ± 0.94	7.16 ± 0.43
EA-Gl isomer 4	nd	2.98 ± 0.14	3.85 ± 0.14	2.51 ± 0.02	3.41 ± 0.23	0.29 ± 0.07	2.51 ± 0.09	1.36 ± 0.15	1.14 ± 0.21
Demethyloleuropein	nd	0.03 ± 0.00	0.20 ± 0.01	0.04 ± 0.01	0.54 ± 0.24	nd	0.18 ± 0.02	0.19 ± 0.01	nq
Rutin	nd	0.18 ± 0.01	0.29 ± 0.04	0.20 ± 0.01	0.28 ± 0.03	0.27 ± 0.02	0.47 ± 0.17	0.53 ± 0.10	0.42 ± 0.05
Luteolin rutinoside	nq	0.05 ± 0.01	nd	0.06 ± 0.01	nq	0.19 ± 0.03	nd	nq	0.10 ± 0.09
Verbascoside	nq	nq	0.23 ± 0.04	nq	nq	nq	0.20 ± 0.11	1.38 ± 0.27	0.62 ± 0.18
Luteolin glucoside isomer 1	3.63 ± 0.01	2.50 ± 0.03	1.74 ± 0.54	1.79 ± 0.32	1.91 ± 0.01	2.53 ± 0.14	2.12 ± 0.65	1.48 ± 0.24	2.57 ± 0.22
Oleuropein glucoside	0.37 ± 0.02	0.20 ± 0.01	0.13 ± 0.08	0.85 ± 0.05	0.02 ± 0.01	nq	0.10 ± 0.01	0.18 ± 0.02	0.57 ± 0.6
Diosmin isomer 1	nd	nq	nd	nq	nq	nq	nq	nd	nq
Apigenin rutinoside	nq	0.04 ± 0.01	0.02 ± 0.00	0.02 ± 0.01	0.04 ± 0.012	0.15 ± 0.01	0.05 ± 0.01	nq	0.07 ± 0.02
Diosmin isomer 2	nq	nd	nd	nq	nq	nq	nq	nd	0.08
Taxifolin	0.13 ± 0.01	0.14 ± 0.02	0.13 ± 0.01	0.77 ± 0.05	0.12 ± 0.02	1.35 ± 0.31	0.29 ± 0.14	0.60 ± 0.01	2.09 ± 0.31
Apigenin glucoside	0.07 ± 0.01	0.22 ± 0.11	0.12 ± 0.06	0.36 ± 0.08	0.07 ± 0.01	0.58 ± 0.05	0.15 ± 0.02	0.12 ± 0.01	0.41 ± 0.04
Luteolin glucoside isomer 2	1.81 ± 0.02	1.21 ± 0.13	0.82 ± 0.04	0.75 ± 0.15	0.68 ± 0.03	1.08 ± 0.12	1.16 ± 0.04	0.77 ± 0.011	1.11 ± 0.01
Chryseriol glucoside	0.47 ± 0.03	0.33 ± 0.02	0.31 ± 0.08	nd	0.28 ± 0.01	0.34 ± 0.03	0.33 ± 0.01	nq	0.17 ± 0.02
Methoxyoleuropein	13.46 ± 0.15	11.27 ± 0.23	9.54 ± 0.12	7.55 ± 0.10	11.56 ± 0.26	9.20 ± 0.41	12.01 ± 0.10	4.60 ± 0.31	7.52 ± 0.16
Oleuropein isomer 1	25.39 ± 1.68	17.87 ± 0.15	20.25 ± 0.15	23.77 ± 0.22	15.76 ± 0.35	12.41 ± 0.27	15.20 ± 0.19	18.96 ± 0.38	22.87 ± 0.47
Oleuropein isomer 2	5.31 ± 0.31	3.39 ± 0.08	4.92 ± 0.37	nd	3.00 ± 0.28	1.15 ± 0.10	1.99 ± 0.09	4.25 ± 0.24	nd
Oleuropein isomer 3	11.55 ± 0.45	6.62 ± 0.11	12.03 ± 0.14	9.79 ± 0.07	5.56 ± 0.02	2.24 ± 0.15	4.49 ± 0.07	11.00 ± 0.12	5.91 ± 0.07
Ligstroside	0.55 ± 0.02	2.15 ± 0.03	2.00 ± 0.10	5.24 ± 0.07	1.57 ± 0.12	0.21 ± 0.01	0.90 ± 0.02	1.72 ± 0.08	6.83 ± 0.03
Oleuropein aglycon isomer 1	6.84 ± 0.05	2.98 ± 0.05	2.77 ± 0.04	nd	4.10 ± 0.09	2.38 ± 0.10	3.73 ± 0.14	4.02 ± 0.07	3.58 ± 0.04
Luteolin	0.72 ± 0.02	1.60 ± 0.01	0.45 ± 0.03	nd	2.19 ± 0.015	2.23 ± 0.34	2.82 ± 0.09	1.22 ± 0.084	1.08 ± 0.01
Quercetin	0.01 ± 0.00	0.08 ± 0.01	0.06 ± 0.01	0.30 ± 0.02	0.06 ± 0.00	0.74 ± 0.01	0.17 ± 0.03	0.40 ± 0.01	0.20 ± 0.02
Oleuropein aglycon isomer 2	2.57 ± 0.01	nd	nd	nd	nq	nd	nd	1.56 ± 0.12	nd
Apigenin	nq	0.13 ± 0.01	nq	nd	0.25 ± 0.05	0.61 ± 0.012	0.32 ± 0.03	0.08 ± 0.01	0.04 ± 0.01
Diosmetin	nq	0.18 ± 0.01	nd	nd	0.29 ± 0.02	nd	nd	0.04 ± 0.01	nd

nd: not detected; nq: not quantified (under the limit of quantification).

## Supplementary Materials

Supplementary Materials Localization of the olive leaves sampling regions.
