# Supplementary material for: Geographical Characterization of Tunisian Olive Tree Leaves (cv. Chemlali) Using HPLC-ESI-TOF and IT/MS Fingerprinting with Hierarchical Cluster Analysis
Source: J Anal Methods Chem. 2018 Mar 13;2018:6789704. doi: 10.1155/2018/6789704 (PMC5872618; doi:10.1155/2018/6789704)
Supplement: Supplementary Materials — Localization of the olive leaves sampling regions. [file 6789704.f1.docx]

**Supplementary material**

**Localization of the olive leaves sampling regions**
